# Magnitude and determinants of surgical site infecion among women underwent cesarean section in Ayder comprehensive specialized hospital Mekelle City, Tigray region, Northern Ethiopia, 2016

**DOI:** 10.1186/s12884-018-2075-8

**Published:** 2018-12-12

**Authors:** Tsehaynesh Aslake Wendmagegn, Gerezgiher Buruh Abera, Weyzer Tilahun Tsehaye, Kibrom Brhanu Gebresslasie, Berhe Girmay Tella

**Affiliations:** School of Nursing, College of Health Sciences, Mekelle University, Mekelle, Ethiopia

**Keywords:** Women, Cesarean section, Ayder, Hospital, Surgical site infection, Tigray, Northern Ethiopia

## Abstract

**Background:**

Surgical site infection (SSI) is an infection that occurs after surgery within 30 days in the part of the body where the surgery took place. Some of the common symptoms are: drainage of cloudy fluid from the surgical wound, pain or tenderness, localized swelling, redness, and raised body temperature. Lack of data on surgical site infection among women who underwent cesarean section (C/S) initiated us to undertake this paper which is targeted to assess the magnitude and determinants of SSI among women who underwent cesarean section.

**Methods:**

Retrospective document review study design was conducted among mothers who underwent cesarean section in Ayder Comprehensive, Specialized Hospital (ACSH) from September 2014 –January 2016. Mother’s charts were selected using systematic random sampling technique. Data were cleaned using EPI info version 3.5.1 and analyzed using SPSS version 20. Descriptive statistics, Bivariate, and multivariable logistic regression were conducted to summarize the data.

**Result:**

A total of 206 medical records of women who underwent C/S in ACSH comprehensive specialized hospital were reviewed. The mean age was 27 years (+ 5 year). The magnitude of surgical site infection was 11.7%. Premature rupture of membrane (PROM), prolonged labor, rural setting, human immune deficiency Virus, chorioaminities and blood loss showed significant association [(AOR = 8.818 95%CI (21.71–35.816)], [AOR = 16.17, 95%CI (2.850–91.819), [AOR = 5.666,95%CI (1.568–20.483)], [AOR = 6.982,95%CI (1.382–35.269), [AOR = 16.17,95%CI (2.850–91.819)] and [AOR = 0.097,9%CI (0.017–0.569)] respectively.

**Conclusion:**

The magnitude of post C/S infection in this study 11.7%. PROM, prolonged labor, residence, HIV, Chorioaminities and blood loss are considered to be an independent risk factor.

## Background

The surgical site infection (SSI) case was identified using the definition provided by the Center of Disease Control (CDC) United States of America (USA) which states that infection is regarded as SSI if it occurs within 30 days of the procedure and has at least one of the following sign and symptoms: purulent drainage from the wound, pain or tenderness, localized swelling, redness, malodor and fever [1].

Among the obstetrical and gynecological surgeries, cesarean section is the most common which attributes to short time consequences like SSI that in turn increases maternal morbidity, prolongs hospital stay and increase medical costs [2].

Approximately 18.5 million cesarean sections are performed yearly worldwide. About 40% of the countries have C/S rates < 10%, about 10% have C/S rates between 10 and 15%, and approximately 50% have C/S rates of > 15%. Majority of the countries that did 18.5 million C/S were from Africa (68.5%) and remaining (29.6%) were from Asia, Latin America and the Caribbean. Due to the worldwide continuous rise in the incidence of cesarean deliveries, the number of women with surgical site infection is expected to increase. The SSI after cesarean section causes physical, psychological and economic burden to the woman, her family and in the community [3]. The rate of C/S in published series in different areas of Ethiopia ranges 14.23–27.6% [4, 5].

The maternal infection following delivery increases by eight-fold when delivered by cesarean section than vaginal delivery. Based on studies related to SSI following caesarean section, the proportion of the SSI ranges from 3 to 15% [6].

Based on the surgical wound classification, cesarean section is a clean contaminated type of surgery where procedure- related chances of infection are less likely to occur, but infection after cesarean section is still a major problem in our setup. The majority of the problem arises from preventable causes, which, if addressed would significantly reduce rates of wound infection.

Identifying risk factors for post-cesarean surgical-site infections (SSIs) could contribute to a decrease in maternal morbidity.

Only one study has been done in Ethiopia to give a picture of a post-cesarean section SSI. Based on the findings of a study on the Surgical Site Infection done in Jimma University, Ethiopia 2010, the SSI among cesarean section cases was 66 (8.55%) [7]. As infection continues to be a common postoperative complication in the study area, and no previous study has been done, there is a need to assess the magnitude and factors contributing to surgical site infection. Thus, this study aims to assess the magnitude and determinants of SSI following cesarean section.

## Methods

Retrospective medical record review study was conducted among mothers underwent C/S from 2014 to 2016 at ACSH, Mekelle city to determine the magnitude and determinants of SSI following C/S delivery.

Mekelle is the capital city of Tigray region and the largest city in northern Ethiopia, at a distance of 783 km from Addis Ababa. It has a population of more than 323,000 among these populations, there are 110,788 females, 104,758 males, 26,536 under five, 60,998 women in reproductive age (15–49 years) and 78,770 < 15 years age [8]. Mekelle has one public referral hospital (ACSH) and three general hospitals namely (Mekelle Hospital, Quiha Hospital and Northern Command Hospital) which are public government hospitals.

ACSH commenced rendering its referral and non-referral services since 2008 to 8 million people in its catchment areas of the Tigray, Afar and Southeastern parts of Amhara Regional States and currently it provides antenatal, delivery and post natal services. It provides a broad range of medical services to both in and out patients of all age groups. As such, the Hospital can be designated as the most advanced medical facility, by all accounts, in the Northern part of the country and that it stands as the second largest hospital in the nation. It has a total capacity of about 500 inpatient beds in four major departments and other specialty units and 84 beds in obstetrics & gynecology department. The obstetrics & gynecology department has 51 staff (5 gynecologist, 7 residents, 18 midwifery and 21 nurses) and is also used as a teaching hospital for the College of Health Sciences, Mekelle University. Annually, ACSH has 2500 deliveries per year; of this, 660 are C/S deliveries [9]. In ACSH, the medical doctors range from resident one up to senior gynecologist specialists. Routinely used antibiotics are Ceftriaxone (as prophylaxis & treatment), Ampicillin and Metronidazole.

The sample size was calculated using the single population proportion formula based on the following assumptions, 95% confidence interval with a 4% margin of error, the expected proportion of post C/S infection taken from a study done in Jimma university hospital which was (P) 8.5% [7]

$$ {\displaystyle \begin{array}{c}\mathrm{N}=\frac{{\left(\mathrm{Za}{/}_2\right)}^2\mathrm{P}\ \left(1\hbox{-} \mathrm{P}\right)=\mathrm{Za}{/}_2=1.96\kern0.5em \mathrm{at}\kern0.5em \mathrm{CI}\ \mathrm{of}\ 95\%}{{\mathrm{d}}^2}\\ {}\mathrm{N}=\frac{6(1.96)2\kern0.5em 0.85(0.915)\mathrm{N}=187}{(0.04)2}\end{array}} $$

By adding 10% contingency for charts with incomplete information the final sample size was 206.

### Sampling procedure

Within the time of September 2014–January 2016, about 923 charts with C/S were found. Systematic sampling technique was used to select 206 charts with cesarean section from records since September 2014–January 2016 in ACSH. By dividing the source population to sample size the k^th^ has been calculated, which was 4. A random number was selected from 1 up to 4 with the lottery method; which was 2 as the first sample chart. The next sample was taken every k^th^ interval provided that for an incomplete chart, we consider the next to be included.

Charts of mothers who underwent cesarean section elsewhere, then referred to ACSH were excluded.

### Data collection procedure and tool

The data were collected from medical records of the 206 selected charts by trained 2 BSc Nurses using structured check list. The checklist was adapted by reviewing different literature of similar studies done on the magnitude and determinant factors of SSI. In this study, socio-demographic characteristic as well as obstetric history, pre-operative and intra-operative, duration of hospital stay, maternal preexisting medical condition variables were considered. The pre-operative events include (Indication for c/s, Prophylaxis taken, Length of days stay in hospital) and intraoperative events included (type of C/S, duration of operation, estimated blood loss and type of incision).The data collection process was supervised by 1BSc nurse who has previous experience in data collection.

### Data quality control

Data collectors and supervisor were carefully trained about the objective of the study, the meaning of each, alternative answers and blank spaces and when to skip as necessary. The checklist was also carefully designed and prepared in English language and was used for data collection. Before the actual data collection, the checklist was pre tested with 10% similar population at Mekelle hospital. Based on the pre-test, the checklist was assessed for its clarity, completion, and the time necessary. During the actual data collection, a supervisor was in place to oversee all activities, and the collected data were reviewed and checked for completeness and consistency by the principal investigator on a daily basis.

### Data management and analysis

Data were coded, entered, checked and cleaned using Epi info version 3.5.1 and exported to SPSS version 20 statistical software package for analysis. Descriptive statistics were computed to determine the proportion of post cesarean section wound infection. Bivariate and multivariable logistic regression was also computed to assess statistical association between the outcome variable and independent variables. Factors that were ≤ 0.2 significant level in the bivariate logistic regression analysis were considered in the multivariable logistic regression analysis. Odds ratios with 95% Confidence Interval (CI) and *p* value (< 0.05) were computed to assess the presence and degree of statistical association between dependent and independent variables.

### Ethical considerations

Ethical clearance was obtained from Mekelle University College of Health Sciences, Research Review Committee, and written permission was obtained from the Medical Director of ACSH to support the research. All the information obtained from the medical record was held with confidentiality and used only for the intended purpose.

## Results

Two-hundred six medical records were reviewed in this study. The finding of this thesis includes demographic data, patient’s obstetric character**,** operation related factors, and the results of post cesarean section infection and factors that contributing to surgical site infection.

### Socio demographic data

The mean age of study participants was 27 years +standard deviation (SD) =5 and 175 (85%) of the study participant within the age group ranged 20–34. The minimum and maximum age of mothers was (15 & 45 years) respectively. Of the total study participants 139 (67.3%) of women who underwent C/S were from urban areas (Table 1).

**Table 1:** Socio-demographic characteristics of the Women who underwent c/s in Ayder referral hospital, Mekelle, Tigray, Northern Ethiopia, 2016 (*N*=206)

Variables	Category	Post c/s SSI				Total (%)
		Yes		No		
		N	%	N	%	
Age	≤19	0	0%	7	100.0%	7(3.4%)
	20–34	18	10.3%	157	89.7%	175(85%)
	≥35	6	25%	18	75.0%	24(11.6%)
Residence	Rural	14	20%	53	79.1%	67(32.5%)
	Urban	10	7.2%	129	92.8%	139 (67.5%)
Ethnicity	Tigray	22	11.1%	176	88.9%	198(96.1%)
	Amhara	0	0%	3	100.0%	3(1.5%)
	Afar	2	40%	3	60.0%	5(2.4%)

### Obstetric characteristic of respondents

More than half 109 (52%) of study participant were multiparous and 8 (7.3%) of them was developed SSI. Almost all the study participants 196 (95.1%) had antenatal care (ANC) follow up & from those 20 (10.2%) were developed SSI. About 172 (83.5%) of study participants had no history of abortion. 44 (21%) of the participants had a history of previous C/S and 5 (11%) of them developed SSI (Table 2).

**Table 2:** Distribution of indication for cesarean section among women who underwent c/sin Ayder referral hospital, Mekelle, Tigray, Northern Ethiopia, 2016 (*N*=206)

Variables	Category	SSI				Total (%)
		YES		NO		
		N	%	N	%	
Parity	Primipara	10	(12.8%)	68	(87.2%)	78(37.9)
	multi para	8	(7.3%)	101	(92.7%)	109(52.9)
	Grand multi para	6	(31.6%)	13	(68.4%)	19(9.2)
ANC follow up	Yes	20	(10.2%)	176	(89.8%)	196(95.1)
	No	4	(40.0%)	6	(60.0%)	10(4.9)
Gestational age at c/s	Preterm	2	(11.1%)	16	(88.9%)	18(8.8)
	Term	21	(12.4%)	148	(87.6%)	169(82)
	Post term	1	(5.3%)	18	(94.7%)	19(9.2)
PROM	Yes	12	(37.5%)	20	62.5%	32(16.0)
	No	12	(6.9%)	162	93.1%	174(84.0)
Prolonged labor	Yes	17	(25.8%)	49	74.2%	66(32)
	No	7	(5.0%)	133	(95.0%)	140(68)
Chorioaminities	Yes	8	(66.7%)	4	(33.3%)	12(5.8)
	No	16	(8.2%)	178	(91.8%)	194(94.2)
Previous c/s	Yes	5	(11.4%)	39	(88.6%)	44(21.4)
	No	19	(11.7%)	143	(88.3%)	162(78.6)
History of abortion	Yes	4	(11.8%)	30	(88.2%)	34(16.5)
	No	20	(11.6%)	152	(88.4%)	172(83.5)

### Indication for cesarean section

**Fig. 1:**
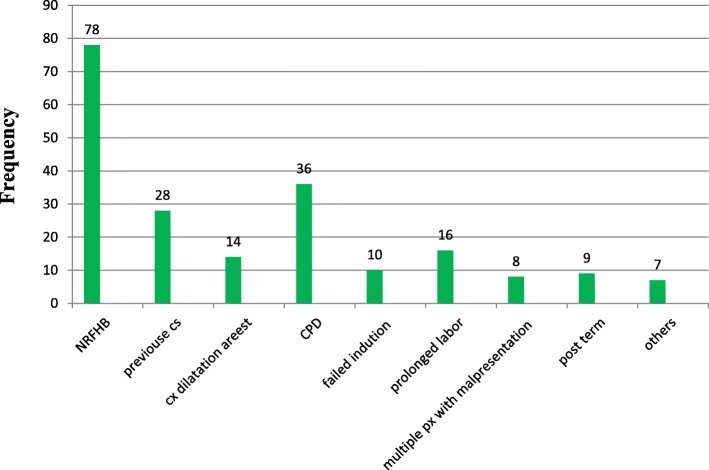
Distribution of indication for cesarean section among women who underwent surgery in Ayder comprehensive specialized hospital, Mekelle, Tigray, Northern Ethiopia, 2016 (*N* = 206)

For 79 (38.2%) of the study participants, the indication for cesarean section was non-reassurance fetal heart beat (NRFHB). For 9 (4.3%), indication was post term (Fig. 1).

### Operation related characteristics

Only 13 (6.3%) of the procedure was elective, and none of these participants developed SSI. About 192 (93.2%) of the study participant had less than 1000 ml intra operative blood loss and only 3% of the skin incision was longitudinal (Table 3).

**Table 3:** Distribution of operation related characteristics among women who underwent C/S in Ayder referral hospital, Mekelle, Tigray, Northern Ethiopia, 2016 (*N*= 206)

Variables	Category	SSI				Total (%)
		YES		NO		
		N %		N	%	
Type of c/s	Elective	0	(0.0%)	13	(100.0%)	13(6.3%)
	Non elective	24	(12.4%)	169	(87.6%)	193(93.7%)
Type of incision	Longitudinal	0	(0.0%)	3	(100.0%)	3(1.5%)
	Transverse	24	(11.65%)	179	(86.89%)	203(98.5%)
Time taken for operation	<30	0	(0.0%)	2	(100.0%)	2(1%)
	30–60	19	(9.8%)	174	(90.2%)	93(93.7%)
	>60	5	(45.5%)	6	(54.5%)	11(5.3%)
Amount of blood loss	<1000	20	(10.4%)	172	(89.6%)	192(93.2%)
	≥1000	4	(28.6%)	10	(71.4%)	14(6.8%)
Length of stay	≤7	18	(9.4%)	173	(90.6%)	191(92.7%)
	>7	6	(40.0%)	9	(60.0%)	15(7.3%)

### Qualification of persons performed cesarean section

**Fig. 2:**
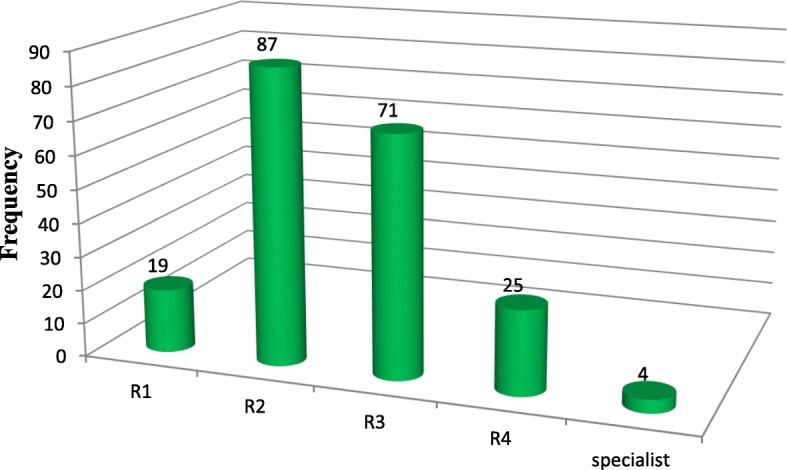
Qualification distribution of person who perform the procedure on women who underwent c/s in Ayder comprehensive specialized hospital, Mekelle, Tigray, Northern Ethiopia, 2016(*N* = 206)

For 87 (42%) of women who underwent cesarean section, the procedure was performed by Resident two (R_2_) (second year resident in 4 year obstetrics and gynecology training program). Four cesarean sections (1.9%) were performed by senior specialists (obstetrics and gynecology specialist) (Fig. 2).

### Magnitude SSI

**Fig. 3:**
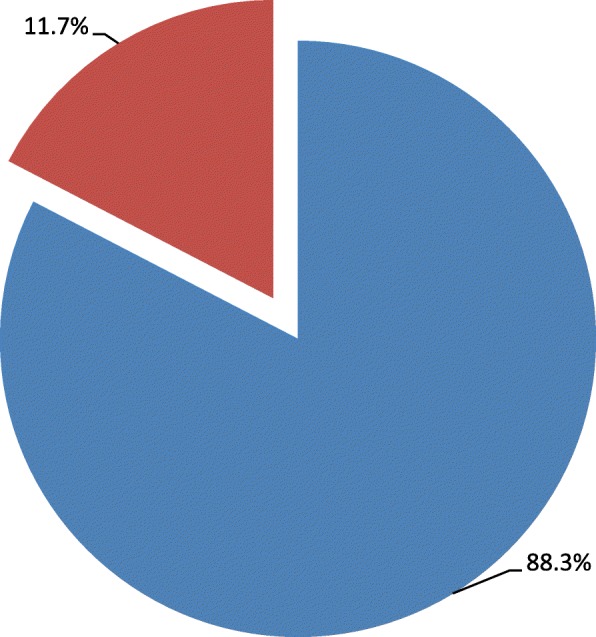
Magnitude of post c/s infection among women who underwent c/s infection in Ayder comprehensive specialized hospital, Mekelle, Tigray, Northern Ethiopia, 2016, (*N* = 206). Legend: With SSI  and  with no SSI

From the total of 206 study participants who underwent cesarean section in Ayder Comprehensive Specialized Hospital, 24 (11.7%) of them had post C/S infection (Fig. 3).

### Preexisting medical condition

The minimum and maximum preoperative hemoglobin label was 8.2 mg/dl-16 mg/dl, respectively, with their mean 13 mg/dl &SD 1.4. About 183 (88.8%) of the study participant were non-reactive for HIV. More than 95% (203) of study participant had no history of diabetes mellitus (DM) (Table 4).

**Table 4:** Frequency distribution of pre-existing medical condition of women who underwent c/s in Ayder referral hospital, Mekelle, Tigray, Northern Ethiopia, 2016 (*N*=206)

Variables	Category	SSI				Frequency	Percent (%)
		Yes		No			
		N	(%)	N	(%)		
Anemia	Yes	4	(25%)	12	(75%)	16	7.8%
	No	20	(10.5%)	170	(89.5%)	190	92.2%
Hypertension	Yes	3	(16.7%)	15	(83.3%)	18	8.7%
	No	21	(11%)	167	(89%)	188	91.3%
DM	Yes	2	(66.7%)	1	(33.3%)	3	1.5%
	No	22	(10.8%)	181	(89.2%)	203	98.5%
HIV	Reactive	5	(41.7%)	7	(58.3%)	12	5.8%
	Non-reactive223	17	(9.3%)	166	(90.7%)	183	88.8%
	Unknown status	2	(18%)	9	(82%)	11	5.3%
Immunosuppressive drug	Yes	0	(0%)	2	(100%)	2	1%
	No	24	(11.8%)	180	(88.2%)	204	99%
Pre op Hgb label	<11 mg/dl	3	(13%)	20	(87%)	23	11.2%
	11mg/dl	21	(11.5%)	162	(88.5%)	183	88.8%

### Determinants of post caesarean section infection

#### Bivariate logistic regression

During bivariate logistic regression analysis, those variables which had ≤0.2 significance level were considered in multivariable logistic regression. Out of twenty-five independent variables, thirteen variables, namely age, residence, anemia, and ANC follow up. Parity, chorioaminities, PROM, prolonged labor, blood loss, length of hospital stay, DM, HIV and duration of operation had shown an association in bivariate analysis with *p*-value less than 0.2, and those variables were entered into multivariable analysis.

#### Multivariable logistic regression

After entering the 13 variables in the multivariable logistic regression analysis simultaneously, Rural setting, PROM, prolonged labor chorioaminities, HIV and blood loss had shown a significant association with *p* value< 0.05.

Accordingly, women who were from rural area were 5.6 times more likely to develop post C/S infection than those from an urban area [AOR = 5.666, 95%CI: (1.568–20.483, *p* = 008)].

The odds of developing post c/s infection among women who had a history of PROM were 8.8 times higher than those who had not [(AOR = 8.818, 95%CI: (21.71–35.816, *p* = 0.002)].

Furthermore, women who had chorioaminities were sixteen times [(AOR = 16.17, 95%CI: (28.50–91.819, *p* = 0.002)] more likely to have post C/S infection.

Women who had prolonged labor were six times [(AOR = 6.064, 95%CI: (1.676–21.949, *p* = 0.006)] more likely to develop post C/S infection. Similarly, HIV positive women are seven times [(AOR = 6.982, 95%CI: (1.382–35.269, *p* = 0.019)] more likely to have post c/s infection than HIV negative women.

Finally, those women who had blood loss less than 1000 ml were almost 90% [(AOR = 0.097,95% CI: (0.017–0.569, *p* = 0.01)] less likely to have post C/S infection than those women who had blood loss greater than 1000 ml (Table 5).

**Table 5:** Bivariate and Multivariable logistic regression analysis of post c/s infection among women underwent C/S in Ayder referral hospital, Mekelle, Tigray, Northern Ethiopia, 2016 (*N*=206)

Variable	Category	SSI		COR (95%CI)	AOR (95% CI)
		YES	NO		
Residence	Rural	14	53	3.408( 1.424-8.152)*	5.666(1.568-20.483)**
	Urban	10	129	1	1
ANC follow up	Yes	20	176	0.199(0.044-0.656)*	0.220(0.2-2.4)
	NO	4	6	1	1
Parity	Primi	10	68	1.857(0.697-4.943)	1.967(0.535-7.230)
	Multi	8	101	1	1
	Grand multi	6	13	5.827(1.745-19.459)*	9.554(1.519-60.075)**
PROM	Yes	12	20	8.1(3.211-20.432)*	8.818(2.171-35.816)**
	No	12	162	1	1
Prolonged labor	Yes	17	49	6.592(2.577-16.861)*	6.064(1.676-21.949)**
	No	7	133	1	1
Chorioaminities	Yes	8	4	22.25(6.035-82.026)*	16.17(2.850-91.819)**
	No	16	178	1	1
HIV	Reactive	5	7	6.975(1.995-24.38)*	6.982(1.382-35.269)**
	Non reactive	17	166	1	1
	Unknown	2	9	2.17(0.433-10.871)	6.979(0.58-83.908)
Blood loss	<1000	22	169	0.26(0.081-0.832)	0.097(0.017-0.569)**
	>=1000	5	10	1	1

*p-value <0.05 in bivariate analysis **p-value <0.05 in multivariable analysis

## Discussion

Medical records were reviewed to find out the magnitude and associated factors of post-cesarean section infection (S.S.I) on women who underwent C/S ACSH. A total of 206 (193 non-elective and 13 elective) were eligible for analysis.

This study indicated that 24 (11.7%) of the study participant had SSI. This figure is higher than the result reported from different studies which revealed that the rate of SSI in US hospitals was 4.1%, in New Zealand 5%, in Brazil 1.44%, China 0.7%, Pakistan 5.8%, Estonian university 6.2%, Saudi Arabia 4–2.4%, and in Nizwa, Oman 2.66%. This discrepancy could be due to the difference in socioeconomic status and health care delivery system [10–13, 14–17].

Similarly, in this study the magnitude of SSI has also shown higher from the studies conducted in England, Nigeria and Jimma which, the rate of post C/S infection was 9.6, 9.3 and 8.55% respectively [7, 18, 19]. This variation might be due to the difference in study area and study participants. However, the results are approximately similar to those from studies conducted in Dhulikhel Hospital, Nepal and Tanzania in which the rates of post c/s infection were 12.6 and 10.9% respectively [20, 21].

This study found a number of factors associated with SSI following C/S. Women who had premature rupture of membrane are six times more likely to develop post C/S infection than those who had not. [AOR = 6.064, CI: (1.676–21.949)]. This finding is in line with the study conducted in US hospitals [OR = 3.0; 95% CI, (3.24–3.5)] and Nigeria (OR = 4.45) [10, 19]. This association could be due to the fetal membrane acting as a barrier to infection. However, when the fetal membrane is ruptured early, it loses its protective effect and microorganisms found in the vagina may easily migrate. This may expose the mother for SSI. This finding was also supported by other studies conducted in Brazil, China, Nepal and Sub-Saharan Africa [12, 13, 20, 22].

The other factor that showed significant association were women who had chorioaminities was sixteen times more likely to have SSI following c/s than those who had not. [AOR = 16.17, 95%CI: (28.50–91.8190] This is also consistent with a study conducted in an Estonian university hospital revealing that women who had chorioaminities were 8.8 times more likely to have post C/S infection (OR, 8.8; 95%CI, 1.1–69.6) [15]. This variation might be related to socioeconomic difference between the countries. Another study conducted in Jimma as also shown significant association [7].

In this study, a rural setting has shown significant association. Women from rural areas were five times more likely to have post C/S infection than those women from urban areas. [(AOR = 5.666, 95% CI: 1.568–20.483)]. This could be due limited access to hygienic materials and poorer hygiene for women living in rural areas when compared to those in urban areas. However, a study conducted in a US urban setting showed significant association with post C/S infection [10].

This study also found that prolonged labor prior to surgery had shown a significant association with SSI following C/S. Women who had prolonged labor were six times more likely to have SSI than those who had not. Similar finding were also reported from a study conducted in New Zealand [11]. This relationship between duration of labor and SSI may be due to increased exposure time where infection can be acquired and to the fact that as duration of labor increase, number of vaginal examinations also increased. Studies conducted in Brazil, China and Tanzania [12, 13, 21] shown that post C/S infection had significant association with frequent vaginal examination.

The other factor that showed significant association was blood loss. Women with intra-operative blood loss of less than 1000 ml were 90% less likely to have SSI than those who had blood loss of more than 1000 ml [(AOR = 0.097,95% CI: 0.017–0.569)]. This finding is consistent with a study conducted in US revealing that women with excessive blood loss were 2.4 times [(OR, 2.42; 95% CI, 2.34–2.49)] more likely to develop post C/S infection [10]. This finding is also supported by a study conducted in Jimma [7]. This association could be due to excessive blood loss associated with poor control and tissue damage due to prolonged retraction, leading to use of extra sutures which may reduce local resistance mechanism.

Finally, the HIV status of women had shown a significant association. HIV positive women had about seven times more likely to have post C/S infection than those HIV negative women [(AOR = 6.982, CI: 1.382–35.269)]. However, other studies did not show its association.

In this study, the age of the mother, ANC follow up, parity, emergency C/S, skin incision, duration of operation, and skill of the person who performs the procedure had no significant association with post c/s infection. However, it is difficult to conclude that those variables are not totally important.

## Conclusions

The magnitude of post C/S infection in this study is high. PROM, chorioaminities, prolonged labor, HIV status, residence and blood loss had shown significant association with post C/S infection.

In this study age of the mother, ANC follow up, parity, emergency c/s, skin incision, duration of operation, and skill of the person who perform the procedure had no significant association with post C/S infection. However, it is difficult to conclude that those variables are not totally important. A small sample was there to show statistical significance.

Based on the finding, the following recommendations are forwarded:

- ❖ Increased awareness, development, and strict implementation of protocol should be done by all the health care professionals in order to minimize and prevent the infection rate after cesarean section
- ❖ Post-operative antibiotics should be given to patients who underwent cesarean section, especially those who are at risk of post cesarean section infection i.e. sero positive, PROM, chorioaminities
- ❖ Evaluate and improve pre, intra and post-operative care through further training and supervision involving more qualified person to decrease post caesarean section infection.
- ❖ Infection prevention procedure in order to reduce the risk of wound infection should be followed appropriately
- ❖ Further prospective study with a large sample size should be conducted.

## Abbreviations

ACSH: Ayder comprehensive, specialized hospital
AOR: Adjusted odds ratio
C/S: Cesarean section
CI: Confidence interval
DM: Diabetes mellitus
OR: Odds ratio
PROM: Premature Rapture of Membrane
SSI: Surgical site infection
